# The genetics and hormonal basis of human gender identity

**DOI:** 10.20945/2359-4292-2024-0232

**Published:** 2024-11-06

**Authors:** Rafael Loch Batista, Luciana Mattos Barros Oliveira

**Affiliations:** 1 Universidade de São Paulo Faculdade de Medicina Departamento de Clínica Médica São Paulo SP Brasil Unidade de Endocrinologia do Desenvolvimento, Laboratório de Genética Hormonal e Molecular (LIM/42), Divisão de Endocrinologia, Departamento de Clínica Médica, Faculdade de Medicina, Universidade de São Paulo, São Paulo, SP, Brasil; 2 Universidade de São Paulo Instituto do Câncer do Estado de São Paulo São Paulo SP Brasil Unidade de Endocrinologia, Instituto do Câncer do Estado de São Paulo, Universidade de São Paulo, São Paulo, SP, Brasil; 3 Universidade Federal da Bahia Instituto de Ciências da Saúde Salvador BA Brasil Instituto de Ciências da Saúde, Universidade Federal da Bahia, Salvador, BA, Brasil

**Keywords:** Gender identity, transgender, sex steroids, genetics, sex development

## Abstract

Gender identity refers to one's psychological sense of their own gender. Establishing gender identity is a complex phenomenon, and the diversity of gender expression challenges simplistic or unified explanations. For this reason, the extent to which it is determined by nature (biological) or nurture (social) is still debatable. The biological basis of gender identity cannot be modeled in animals and is best studied in people who identify with a gender that is different from the sex of their genitals such as transgender people and people with disorders/differences of sex development. Numerous research studies have delved into unraveling the intricate interplay of hormonal, neuroanatomic/neurofunctional, and genetic factors in the complex development of core gender identity. In this review, we explore and consolidate existing research that provides insights into the biological foundations of gender identity, enhancing our understanding of this intriguing human psychological trait.

## INTRODUCTION

Sexual differentiation involves the development of distinctions between males and females, a phenomenon observed widely in nature, including in human biology (1,2). A notable sexually dimorphic trait in humans is gender identity (3,4), defined as an individual's intrinsic perception of themselves as female, male, or as a gender alternative to conventional male and female classifications (4). In cisgender individuals, the gender identity aligns with the gender assigned at birth and remains consistent throughout their lifespans. Conversely, transgender individuals may consistently or intermittently identify with a gender different from the one assigned at birth (5-7).

Given the intricate nature of this framework and its clinical implications, significant attention has been dedicated to understanding the origins of the sexual differentiation process (4,8-10). The literature engages in a substantial debate on factors, whether related to nature or nurture, contributing to the sexual differentiation of the brain (11). Nevertheless, it is firmly established that biology plays a pivotal role (12). Accumulating evidence suggests that prenatal sex hormones exert a lasting impact on human sexual development, and heritability studies suggest the involvement of genetic components (13,14).

**Figure 1:**
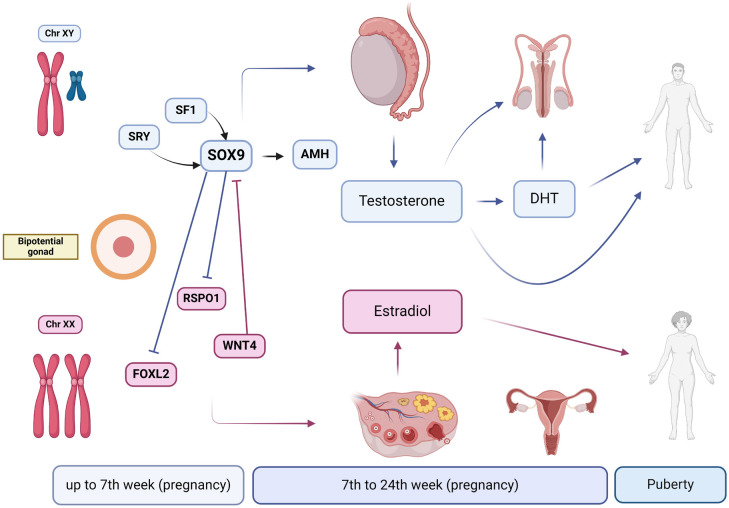
Pathways of human gonadal sexual development. Up to the 7th week of pregnancy, the gonad remains undifferentiated, possessing the potential to develop into either testes or ovaries. The differentiation process is driven by specific genetic and hormonal signals. In the presence of the Y chromosome, the sex-determining region Y (*SRY*) gene is expressed. Subsequently, *SRY* initiates the male developmental pathway by promoting the expression of SOX9, a critical transcription factor. Activation of SOX9 is further supported by steroidogenic factor 1 (SF1). Once activated, SOX9 induces the production of anti-Müllerian hormone by Sertoli cells in the developing testes, which plays a vital role in inhibiting the development of female internal genitalia by causing the regression of Müllerian ducts. Concurrently, Leydig cells in the testes begin producing testosterone under the influence of SOX9. Testosterone is essential for the development of male internal genitalia. Additionally, testosterone is converted to dihydrotestosterone, which is crucial for the formation of male external genitalia and the development of secondary sexual characteristics during puberty. In the absence of the *SRY* gene, the female developmental pathway is initiated. Factors such as RSPO1 and WNT4 support ovarian development by inhibiting SOX9 and promoting the differentiation of the gonad into ovaries. Along with RSPO1 and WNT4, FOXL2 plays a significant role in supporting ovarian development and function. The developing ovaries produce estradiol, a crucial hormone for the development of female reproductive organs and secondary sexual characteristics. The hormonal influence continues from embryonic stages through puberty, shaping the development and function of the reproductive system.

Human sexual development is a dynamic process regulated by genes and executed by endocrine mediators in the form of steroids and peptide hormones (1). The first stage of sexual development is determined by chromosomal sex (presence of the X or Y chromosome) (Figure 1). This chromosome will influence the determination of gonadal sex, differentiating the bipotential gonad into ovaries or testes (15). The presence and expression of the *SRY* gene (located on the distal portion of the short arm of the Y chromosome) direct gonadal differentiation toward testes, forming Leydig and Sertoli cells. Sertoli cells produce anti-Müllerian hormone, which causes the involution of Müllerian derivatives, while Leydig cells produce testosterone, differentiating Wolffian ducts into vas deferens, epididymis, and seminal vesicles. The conversion of testosterone to dihydrotestosterone by the action of 5α-reductase type 2 occurs between the sixth and twelfth weeks of gestation and is essential for the development of male internal genital organs and the virilization of external genitalia (16).

Sexual development continues after gonadal differentiation with cerebral sexual differentiation, occurring in the second half of gestation, where gonadal steroids (especially testosterone) act, even in the prenatal period, causing organizational effects on the brain, leading to permanent changes in brain structure and sexual behavior (17). Later, during puberty, with gonadal hormonal production, these prenatally organized cerebral circuits are reactivated, causing what is defined as brain activation effects of sexual steroids, maturing and completing human sexual development (17,18).

Cerebral sexual development follows the same dynamics as gonadal development, where the presence of androgens is necessary for male development (19). All these orchestrated dynamics are necessary for human psychosexual development, of which gender identity is one of the pillars.

**Figure 2:**
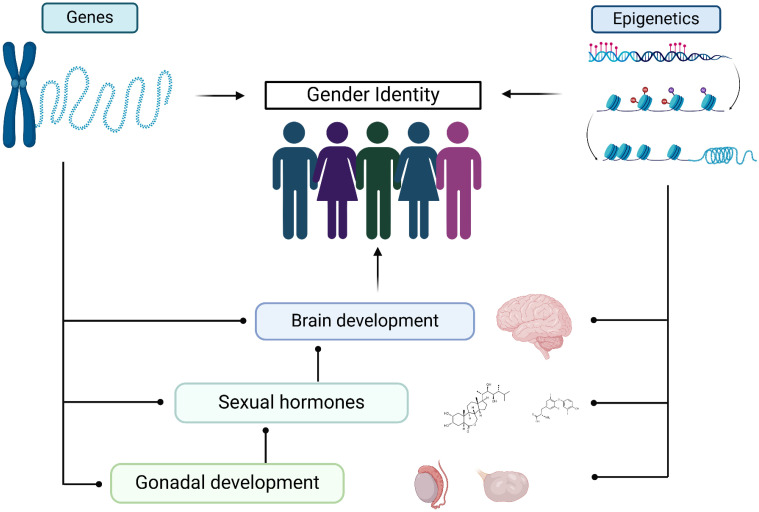
The biological influencers of gender identity.

Gender identity can arise from a complex interplay between nature (biology) and nurture (social) (Figure 2). This review synthesizes evidence that underscores the biological underpinnings of gender identity development in humans.

## THE GENETICS OF GENDER IDENTITY

To investigate the genetics of gender identity, a study examined variants in 12 sex signaling genes, such as *COMT, CYP11A1*, *HSD17B6, STS*, and *SULT2A1*, in a cohort of 380 Caucasian (non-Latino) transgender women and compared the variants with those in 344 white male control subjects without gender dysphoria. The authors identified a unique link between TA repeats in *ERα* and gender dysphoria. While some previously reported associations were not replicated, interactions between genes like *AR, ERβ*, and others were highlighted (20).

Indeed, the exploration of DNA polymorphisms in genes such as *ERβ, ERα*, *AR*, and aromatase (*CYP19A1*) has been undertaken to understand their involvement in gender identity. However, reported results have been inconsistent or negative, potentially due to small sample sizes and the heterogeneous nature of the transsexual population. Addressing this, a Spanish study investigated the implications of specific polymorphisms (CA)n-ERβ (rs113770630), XbaI-ERα (rs9340799), (CAG)n-AR (rs193922933), and (TTTA)n-CYP19A1 (rs60271534) in a large and homogenous sample comprising 549 early-onset androphilic male-to-female individuals *versus* 728 male controls and 425 early-onset gynephilic female-to-male individuals *versus* 599 female controls (21). Utilizing a binary logistic regression model, the study concluded that certain allele and genotype combinations of *ERβ, ERα*, and *AR* are implicated in the genetic basis of transgender identity. Specifically, male-to-female gender development requires AR, accompanied by ERβ, with an inverse allele interaction observed between *ERβ* and *AR* in the male-to-female population. Additionally, *ERβ* and *ERα* are associated with male gender identity in the female-to-male population, although no interaction between the polymorphisms was found. These findings underscore the significant role of *ERβ* in human brain differentiation (21).

In their search for genetic variants in transgender individuals, Theisen and cols. conducted whole exome sequencing on the genomic DNA of 13 transgender males and 17 transgender females, identifying 120,582 genetic variants. Following filtering, 441 variants in 421 genes were retained for further analysis, encompassing 21 nonsense, 28 frameshifts, 13 splice-region, and 225 missense variants. Notably, 21 variants in 19 genes were found to be associated with pathways involved in brain sexual differentiation (*AKR1C3, BOK*, *CDH8, CDK12*, *CTNNA2, DNER*, *DSCAML1, EGF*, *EFHD2, GRIN1*, *KCNK3, MAP4K3*, *PIK3CA, PPARGC1B*, *RIMS3, RIMS4*, *SPHK1, SYNPO*, and *TNN*) or the estrogen/estrogen receptor pathway (*AKR1C, CDK12*, *PIK3CA, PPARGC1B*) (22).

## HERITABILITY STUDIES IN GENDER DYSPHORIA

Heritability studies offer valuable insights into the genetic components of various conditions or biological traits, with scores measured from 0 to 1. A score of 0 indicates no genetic influence, while 1 signifies complete genetic determination. Gender dysphoria has been primarily explored through twin studies, with limited investigations into family studies (23).

A study involving 1,891 twins reported heritability patterns of 0.50-0.57 in men and 0.30-0.37 in women for childhood gender dysphoria. However, the potential for recall bias should be considered, given the twins’ median age of 29 years. Subsequent research has shown varied heritability estimates, such as one study suggesting genetics account for 62% of the variance in gender dysphoria. Some studies found an inheritance pattern of 0.21 in boys and 0.74 in girls, while another study identified a female inheritance pattern of 0.11, indicating a very low influence of genes (24,25). Nevertheless, caution is warranted due to the potential for errors, and none of these studies specifically delved into examining individual genes associated with gender dysphoria.

Regarding twins, Heylens and cols. collected previously reported data from twins presenting gender dysphoria and found a concordance rate of 39.1% in monozygotic twins and no concordance (0%) in dizygotic pairs (26). A separate investigation of gender dysphoria observed concordance rates of 33% for assigned males and 23% for assigned females among same-sex dizygotic pairs (27). In a thorough investigation conducted in Sweden, the analysis of gender dysphoria in twins utilized a register-based population spanning from 2001 to 2016. The primary outcome focused on individuals with at least four gender dysphoria diagnoses or at least one diagnosis followed by gender-affirming treatment (28). Among the 2,592 full siblings registered as relatives of gender dysphoria cases, 67 were twins, with probands’ ages at first gender dysphoria diagnosis ranging from 11.2 to 64.2 years. Notably, the percentage of different-sex twins presenting with gender dysphoria (37%) significantly exceeded that in same-sex twins (0%, p < 0.001) and non-twin sibling pairs (0.16%).

Expanding beyond twin studies, a Spanish study on 995 consecutive transsexual probands identified 12 pairs of gender dysphoria in non-twin siblings (29). This study further revealed that the likelihood of a sibling being transsexual was 4.48 times higher for siblings of male-to-female transsexual probands than for siblings of female-to-male transsexual probands, and 3.88 times higher for the brothers than for the sisters of transsexual probands.

## EPIGENETICS AS A BASIS FOR GENDER INCONGRUENCE

Epigenetics explores how external factors influence gene expression and phenotype without altering the underlying DNA sequence, shedding light on how environmental cues shape biological traits across generations (30).

Considered one of the most enduring and extensively researched epigenetic modifications, DNA methylation entails adding a methyl group to cytosine residues adjacent to guanine in DNA, specifically at CpG sites (31). This process is closely tied to alterations in gene transcription, particularly when occurring within gene promoter regions (31). Through the DNA methylation analysis, researchers have illuminated the pivotal role of epigenetic regulation in governing the sexual differentiation of the brain (32,33). This dynamic field holds potential as a mechanism influencing gender development, given its sensitivity to environmental stimuli (34).

Nugent and cols. elegantly proposed the involvement of epigenetics in sexual development through a sophisticated experimental approach. Utilizing mice, the researchers demonstrated that gonadal steroids primarily diminish the activity of DNA methyltransferase (Dnmt) enzymes in the highly sexually dimorphic pre-optic area, leading to a reduction in DNA methylation and the liberation of masculinizing genes from epigenetic repression. Pharmacological inhibition of Dnmts replicated the effects of gonadal steroids, eliciting masculinized neuronal markers and male sexual behavior in females. Moreover, the conditional knockout of the *de novo* Dnmt isoform, Dnmt3a, also led to the masculinization of sexual behavior in female mice. Subsequent RNA sequencing unveiled gene and isoform variants influenced by methylation that could contribute to the distinct reproductive behaviors observed between males and females, ultimately suggesting that the active suppression of masculinization via DNA methylation sustains brain feminization (35). Additionally, inhibiting DNA methylation in developing mouse brains has been shown to result in abnormal neurobehavioral profiles and disrupt sexually dimorphic neurobehavioral phenotypes in adulthood (36).

To analyze global DNA methylation in a population with gender incongruence before gender-affirming hormone treatment, Ramirez and cols. conducted a global CpG (cytosine-phosphate-guanine) methylation analysis on blood samples from 16 transgender individuals before gender-affirming hormone treatment and 16 cisgender individuals (34). The results showed that both populations (cis and trans) differed in the degree of global CpG methylation before gender-affirming hormone treatment. The most significant CpGs were related to genes *WDR45B, SLC6A20*, *NHLH1, PLEKHA5*, *UBALD1, SLC37A1*, *ARL6IP1, GRASP*, and *NCOA6*. They also found that trans men and trans women share a CpG related to the *MPPED2* gene. The enrichment analysis showed that these genes involve diverse functions, including the central nervous system and brain development. The authors identified two global CpG methylation profiles in cis and trans populations before gender-affirming hormonal therapy, supporting the hypothesis that epigenetics plays a role in gender incongruence.

## EVIDENCE OF THE INFLUENCE OF PRENATAL SEX STEROIDS ON GENDER IDENTITY

In the early stages of development, testosterone plays a crucial role in shaping the mammalian brain's sexual differentiation, leaving lasting impacts on behavior (17). In humans, testosterone levels rise in males from approximately weeks 8 to 24 of gestation and resurface during early postnatal development (mini puberty) (17,37). Individuals exposed to atypical concentrations of testosterone or other androgenic hormones during prenatal stages consistently exhibit heightened male-typical juvenile play behavior, variations in sexual orientation and gender identity, and an increased propensity for physically aggressive behavior (38,39). The influence of prenatal androgen exposure in facilitating a male gender behavior has been widely established, yet its impact on gender identity is not as clear (40). This is mainly due to the heterogeneity of studies resulting from the absence of standardized tools for assessing gender identity (41). However, studies exploring additional behavioral outcomes following substantial prenatal androgen abnormalities are either limited in sample size or exhibit inconsistent results, thus lacking equally conclusive evidence.

Conditions related to prenatal androgen exposure have been a model for studying the influence of sex steroids on gender behavior. Differences/disorders of sex development (DSD) is a collective term for a group of relatively rare congenital conditions associated with an alteration in chromosomal, gonadal, or anatomic sex (41,42). Some individuals with DSD exhibit a 46,XY chromosome complement, collectively known as 46,XY DSD (41-43). Those with 46,XY DSD may display varying degrees of virilization in their external genitalia, along with variable development of structures derived from the Wolffian and Müllerian ducts (41,43). Regardless of the extent of undervirilization, the root cause of 46,XY DSD can be attributed to (A) decreased production of androgens such as testosterone or dihydrotestosterone during fetal sex differentiation, (B) impaired androgen action at target tissues throughout life, or (C) alterations in the testosterone metabolism (43,44). All these conditions result in a 46,XY fetus with varying degrees of undermasculinization. In many 46,XY DSD-related conditions, some degree of genital virilization is present, suggesting a certain level of cerebral virilization (45-47). Hence, gender outcomes have been studied in individuals with these conditions.

Among individuals with a 46,XX karyotype, DSD conditions result from elevated androgen exposure during either prenatal or postnatal periods (48). The excess androgens in 46,XX patients can originate from fetal (gonadal or adrenal), placental, or maternal sources (49). Congenital adrenal hyperplasia (CAH) is the primary cause, accounting for 90%-95% of cases. Due to androgen excess, DSD 46,XX can also be prenatally androgen-exposed (50).

In 46,XX infants with CAH, the sex assigned at birth may influence gender identity outcomes (50,51). Despite experiencing varied levels of prenatal and postnatal androgen exposure, prenatal androgen exposure in CAH has been linked to male gender role behavior during childhood, but not to male gender identity in adulthood (52-55). Research indicates that most 46,XX individuals with CAH develop a female gender identity (56,57). Berenbaum and Bailey found no correlation between the severity of external genital masculinization and the prevalence of male gender identity or gender dysphoria (55). They cautioned against assuming that gender identity is primarily determined by prenatal androgen exposure, suggesting that the relationship between genital masculinization and brain masculinization may not be proportional (55). Similarly, Meyer-Bahlburg and cols. observed masculinized gender behavior without gender dysphoria in girls aged 5-12 years with CAH, emphasizing the intricate connection between androgen exposure and gender identity development (58). Notably, gender behavior studies among individuals with CAH found no correlation between gender identity and either the severity of the CAH condition or the degree of genital virilization. However, the reported rate of gender dysphoria and/or male identification in CAH females is 5%, surpassing the prevalence of gender dysphoria in the general population (58,59).

A direct association between male gender identity and prenatal androgen exposure has been documented in 46,XY individuals with DSD conditions involving prenatal androgen exposure, such as 5α-reductase type 2 deficiency and 17β-hydroxysteroid dehydrogenase type 3 deficiency since both conditions exhibit a high prevalence of male gender identity and male gender role behavior (40,46,60,61).

In brief, 5α-reductase is the crucial enzyme for synthesizing dihydrotestosterone from testosterone (61,62). In fetuses lacking 5α-reductase, the conversion of testosterone to dihydrotestosterone does not occur during the critical period of external genitalia differentiation (63). Since dihydrotestosterone is essential for external genital virilization, their genitalia appear typically female or only mildly masculinized at birth (64). However, individuals with 5α-reductase type 2 deficiency still produce and respond to testosterone in a manner similar to unaffected males. They undergo virilization during puberty if their testes remain in place, subject to the effects of prenatal testosterone exposure (16,63).

The enzyme 17β-hydroxysteroid dehydrogenase 3 is responsible for synthesizing testosterone from the precursor hormone androstenedione. Notably, 46,XY individuals with 17β-hydroxysteroid dehydrogenase 3 deficiency cannot convert androstenedione to testosterone, resulting in female-typical or mildly masculinized external genitalia at birth (16,63,65).

Both conditions exhibit a high prevalence of gender dysphoria. The frequency of gender change is notably higher among 46,XY individuals with DSD compared with the general 46,XY population (20,35). However, a greater incidence of gender transition is observed in cases of 5α-reductase type 2 and 17β-hydroxysteroid dehydrogenase 3 deficiencies (66). Nevertheless, gender dysphoria is not exclusive to these specific 46,XY DSD diagnoses among 46,XY people with DSD.

The manifestation of male gender identity in 46,XY individuals, despite female sex assignment and female-like external genitalia at birth, supports the role of androgens in shaping gender identity. The impact of prenatal androgen exposure has been investigated through studies on the general population using indirect measures, such as finger ratio, defined as the length of the index finger to the ring finger (67). The finger ratio is often considered a potential marker of prenatal androgen levels, where lower 2D:4D (2D = index finger; 4D = ring finger) levels suggest high prenatal testosterone and low estrogen, while higher 2D:4D indicates the opposite (41,42,67,68). Despite this, research on the association between finger ratio and gender identity has yielded inconsistent results (43). To clarify these inconsistencies, a study enrolled 464 participants to investigate the relationship between gender dysphoria and second-to-fourth digit length ratio (2D:4D), alongside a meta-analysis of 17 prior studies comprising 3,674 participants (69). The findings demonstrated a notably elevated left-hand 2D:4D ratio in male-to-female transgender individuals compared with male controls, consistent across both the primary study and the meta-analysis. Conversely, no significant distinctions were detected in female-to-male transgender individuals when compared with female controls. These results imply a potential association between transgender identity and 2D:4D ratios.

Another indirect indicator of prenatal hormone exposure is otoacoustic emission (OAE), representing the faint sound produced by the auditory transduction apparatus of the inner ear (69,70). Notably, OAEs exhibit gender-based differences, with weaker emissions in newborn males than females, a distinction that persists throughout the lifespan (71). An intriguing study showed that boys with gender dysphoria exhibited stronger, more female-typical click-evoked OAEs (CEOAEs), whereas girls with gender dysphoria showed no difference in emission strength compared with control girls. Assuming CEOAE amplitude reflects relative androgen exposure, its findings suggest that boys with gender dysphoria may have experienced lower levels of androgen during early development compared with control boys (72). To assess the potential impact of postnatal hormonal effects on CEOAEs, a cross-sectional study examined whether interventions such as gonadotropin-releasing hormone analogs (GnRHa) to suppress endogenous sex hormones and pubertal development, followed by cross-sex hormone treatment, could influence CEOAEs in adolescents with gender dysphoria compared with age- and sex-matched controls (73). Sex-typical CEOAE differences were noted in control and treatment-naïve trans boys, but not in other gender dysphoria groups. Treatment-naïve trans girls tended to display more female-typical CEOAEs, indicating undermasculinized early sexual differentiation and supporting a prevailing hypothesis on gender dysphoria etiology. Aligning with androgen suppression effects, trans boys receiving testosterone plus GnRHa exhibited significantly weaker right-ear CEOAEs compared with control girls, with a similar trend observed in GnRHa-only treated trans boys. Unexpectedly, trans girls exhibited CEOAE masculinization with estradiol addition. These findings underscore that CEOAEs may not consistently reflect prenatal androgen exposure, as they can be modulated postnatally by hormonal treatment.

## GENDER IDENTITY AND NEUROANATOMIC/ NEUROFUNCTIONAL DIFFERENCES

Many sex differences in human brains are evident in the sizes of particular brain regions. The caudate nucleus, hippocampus, Broca's area, anterior commissure, and right parietal lobe are larger in females than in males, while the hypothalamus, stria terminalis, and amygdala are larger in males than in females (74). Most sex differences in the brain have been investigated in regions important for sexual function and reproduction, such as the hypothalamus. The influence of gonadal hormones on the sexual differentiation of these structures has been studied extensively, for instance, in the case of the sexually dimorphic nucleus of the preoptic area (74).

There is evidence that sex differences in cortical structure vary in a complex and highly dynamic way across the human lifespan. After studying neonatal brain structure and comparing their findings with existing literature, Knickmeyer and cols. proposed four general patterns: (A) sex differences that are stable across the lifespan, (B) sex differences that are not present in the neonate but arise during childhood and/ or adolescence, (C) sex differences that are present during periods of high circulating gonadal steroids (*e.g.*, neonate and adult, but not childhood), and (D) sex differences that are unique to the neonate. Sexual dimorphism of the brain reflects the dynamic interplay of multiple mechanisms, both biological (*e.g.*, prenatal hormone production, neonatal hormone production, pubertal hormone production, direct sex-chromosome effects) and experiential (*e.g.*, parental expectations and interactive behavior, exposure to physical hazards, culturally influenced lifestyle differences) (74,75).

Ingalhalikar and cols. examined sex differences in a large population of 949 youths by comprehensively analyzing the diffusion-based structural connectomes of the brain. Because the population had a wide age range (8-22 years), they also examined the sex differences during the course of development (74,76). With the aim of identifying at what stage of development these sex differences manifest, they analyzed the population in three groups that aligned with childhood, adolescence, and young adulthood. The connectivity profiles showed an early separation between the developmental trajectories of the two genders, with adolescent and young adult males displaying higher intrahemispheric connectivity and females of the same age displaying higher interhemispheric connectivity. Although the dominance of intrahemispheric connectivity in males was established early on and preserved throughout the course of development, interhemispheric connectivity dominance in females was seen mainly in the frontal lobe during adolescence but was more dispersed across the lobes during adulthood (76). Greater interhemispheric connectivity in females would facilitate the integration of the analytical and sequential reasoning modes of the left hemisphere with the spatial, intuitive processing of information of the right hemisphere. A behavioral study on the entire sample, of which this imaging study is a subset, demonstrated pronounced sex differences, with females outperforming males on attention, word and face memory, and social cognition tests and males performing better than females on spatial processing and motor and sensorimotor speed (76). These differences were mainly observed in mid-adolescence (ages 12-14 years), where males performed significantly faster than females on motor tasks and more accurately on spatial memory tasks (76). They are also consistent with activation studies using functional MRI, which have reported greater interhemispheric activation in females on a language task, in which they excelled, and greater focal intrahemispheric activation in males on a spatial task, in which they excelled (76).

However, Eliot and cols. believe that the human brain is not "sexually dimorphic" because differences between male and female brains are extremely subtle and variable. The term "dimorphism" has potent heuristic value, reinforcing the belief in categorically distinct organs: a "male brain" and a "female brain" that have been evolutionarily shaped to produce two psychologically distinct categories of people. A picture is emerging not of two brain types nor a continuous gradient from masculine to feminine, but of a multidimensional "mosaic" of countless brain attributes that differ in unique patterns across all individuals (77).

**Table 1:** Table of genetic and heritability studies on gender dysphoria

Study Type	Population Details	Findings	Study Reference
Genetic study	380 Caucasian transgender women, 344 controls	Identified unique link between TA repeats in *ERα* and gender dysphoria, interactions between *AR, ERβ*, and others.	20
Genetic study	549 male-to-female individuals, 728 male controls; 425 female-to-male individuals, 599 female controls	Certain allele and genotype combinations of *ERβ, ERα*, and *AR* implicated in genetic basis of transgender identity.	21
Whole exome sequencing	13 transgender males, 17 transgender females	Identified 441 variants in 421 genes, 21 associated with sexual differentiation or estrogen/estrogen receptor pathway.	22
Heritability study (twins)	1,891 twins	Heritability patterns: 0.50-0.57 in men, 0.30-0.37 in women for childhood gender dysphoria.	23
Heritability study (various)	Various populations	Genetics account for 62% of variance in gender dysphoria; inheritance pattern: 0.21 in boys, 0.74 in girls, 0.11 in women.	24, 25
Twin study	Previously reported twins with gender dysphoria	Concordance rate of 39.1% in monozygotic twins, 0% in dizygotic pairs.	26
Twin study	Twins with transgender identity	Concordance rates: 33% for assigned males, 23% for assigned females among same-sex dizygotic pairs.	27
Register-based twin study	Swedish population from 2001 to 2016	Different-sex twins (37%) presented gender dysphoria more than same-sex twins (0%) and non-twin siblings (0.16%).	28
Family study	995 consecutive transsexual probands	Sibling being transsexual 4.48 times higher for male-to-female probands, 3.88 times higher for brothers than sisters.	29

Studying transgender individuals’ brains has been important in advancing our understanding. The observed shift away from a male-typical brain anatomy toward a female-typical one in people who identify as transgender women suggests a possible underlying neuroanatomical correlate for a female gender identity (78). The brain anatomy in the current sample of transgender women is shifted toward their gender identity – an observation that is at least partly in agreement with previous reports. Even though findings are not immediately comparable, all existing structural MRI classifier studies – as well as a recent resting-state functional MRI classifier study – seem to support the notion of a "shift" away from the biological sex toward the gender identity in transgender people (78).

A comprehensive systematic review and meta-analysis examined the relationship between gender-affirming steroid hormonal therapy and cognitive function in transgender young adults and focused mostly on visuospatial ability and verbal memory. The results showed a statistically significant enhancement in visuospatial ability in transgender males following gender-affirming hormone treatment. The pooling of cross-sectional studies showed an improved performance in verbal working memory among treated compared with non-treated transgender female individuals. Gender-affirming hormone administration has no adverse effect on cognitive domains (79).

In conclusion, the exploration of gender identity reveals a multifaceted interplay between biological and social factors, highlighting the complexity of its development. Genetic studies have identified potential links between specific genes and transgender identity, shedding light on the genetic underpinnings of gender identity. However, inconsistencies in results underscore the need for larger, more homogeneous studies. Epigenetic analyses offer further insights, indicating the influence of environmental factors on gene expression, potentially contributing to gender incongruence. Prenatal exposure to sex steroids has been extensively studied, particularly in conditions like DSD, providing evidence of its impact on gender behavior and identity. Neuroanatomic and neurofunctional differences between sexes have been observed, suggesting possible neurobiological correlates of gender identity. Studies on transgender individuals’ brains have revealed structural shifts toward their gender identity, supporting the neurobiological basis of gender identity. Overall, the intricate nature of gender identity is influenced by several biological factors. However, the orchestration of gender identity encompasses multiple rhythms, lacking exclusivity or singularity. Instead, it manifests as a diverse and plural human phenomenon.

**Box 1:** Glossary of gender identity and sexual orientation terms

**Transgender Female:** An individual assigned male at birth who identifies and lives as a female. This term is sometimes abbreviated as trans woman.
**Transgender Male:** An individual assigned female at birth who identifies and lives as a male. This term is sometimes abbreviated as trans man.
**Cisgender:** A term used to describe individuals whose gender identity matches the sex they were assigned at birth. For example, a person assigned female at birth who identifies as female.
**Androphilic:** An adjective used to describe individuals who are sexually attracted to men or masculinity. This term can be used regardless of the individual's gender identity.
**Gynephilic:** An adjective used to describe individuals who are sexually attracted to women or femininity. This term can be used regardless of the individual's gender identity.
**Non-binary:** A gender identity that does not fit within the traditional binary concept of being male or female. Non-binary individuals may identify as a mix of both genders, neither gender, or a different gender altogether.
**Gender Dysphoria:** A psychological condition where an individual experiences distress or discomfort due to a discrepancy between their gender identity and their sex assigned at birth, civil name, or even some physical aspects of their body.
**Gender Identity:** One's internal, deeply held sense of being male, female, a blend of both, or neither. Gender identity can correlate with the sex assigned at birth, or it can differ from it.
**Sexual Orientation:** Refers to the type of sexual, romantic, or emotional attraction one has the capacity to feel for others, typically categorized as heterosexual, homosexual, bisexual, pansexual, asexual, etc.
